# Characteristics related to biomarkers of neutrophil, eosinophil, and mast cell activation

**DOI:** 10.1038/s41598-025-18398-9

**Published:** 2025-09-12

**Authors:** Anna Sivander, Maria Hårdstedt, Elisabet Nerpin, Robert Movérare, Anders Sjölander, Christer Janson, Andrei Malinovschi

**Affiliations:** 1 Department of Medical Sciences, Clinical Physiology, Uppsala University, Uppsala, Sweden; 2 Center for Clinical Research Dalarna, Uppsala University, Uppsala, Sweden; 3 Vansbro Primary Health Care Center, Vansbro, Sweden; 4 School of Medical Sciences, Örebro University, Örebro, Sweden; 5 School of Health and Welfare, Dalarna University, Falun, Sweden; 6 Department of Medical Sciences, Respiratory-, Allergy- and Sleep Research, Uppsala University, Uppsala, Sweden; 7 Thermo Fisher Scientific, Uppsala, Sweden; 8 Laboratoriemedicin Dalarna, Klinisk kemi, Falu lasarett, 791 82 Falun, Sweden

**Keywords:** Biomarkers, Eosinophil, Neutrophil, Mast cell, Disease, Biomarkers, Diseases

## Abstract

Systemic inflammation is important in many medical conditions. Activation markers of inflammatory cells can be quantified, but are less studied. Therefore, we studied individual characteristics and diseases in relation to neutrophil gelatinase-associated lipocalin (NGAL) and myeloperoxidase (MPO), both for neutrophils, eosinophil-derived neurotoxin (EDN), eosinophil cationic protein (ECP), and mast cell tryptase. Spirometry and serum biomarkers were assessed in 498 participants (252 males) aged ≥ 40 years from the Uppsala center of the population-based Burden of Obstructive Lung Disease study. Higher MPO, NGAL and EDN levels related to heart disease. Higher ECP and EDN levels were both related to asthma while higher EDN related to male sex and atopy. Higher tryptase levels were found in subjects with obesity, chronic airflow limitation (CAL), or hypertension. In multivariate analyses, subjects with heart disease had 22.1% (95% confidence interval 4.1%, 43.3%) higher MPO, 19.3% (7.8%, 31.9%) higher NGAL, and 23.2% (3.0%, 47.3%) higher EDN than subjects without heart disease. The association between CAL and tryptase was not consistent after adjustment for age and BMI as continuous variables. The higher EDN levels in heart disease is a novel finding that require further studies to elucidate the clinical importance.

## Introduction

Systemic inflammation is an important process in many diseases, including metabolic, cardiovascular, and respiratory diseases. Different inflammatory cells may be involved and can be assessed through routine tests using differential cell counts. Activation markers of inflammatory cells are also available, but have been studied to a limited extent as regards individual characteristics and disease^1^.

Neutrophil granulocytes are the first line of defense against invasive infections and tissue damage in humans. Granules of various proteins are present in these cells to regulate, e.g., activity and phagocytosis. Primary granules, contain myeloperoxidase (MPO) and are highly toxic upon release. MPO is present in the extracellular environment and the phagolysosomal compartment following neutrophil activation, in both peripheral blood and tissues^2^. MPO also seems to play an important role in the inflammatory response^3^. It has been shown to be a local mediator of tissue injury in several inflammatory disorders, including atherosclerosis, cardiovascular disease, kidney disease, pulmonary inflammation, rheumatoid arthritis, skin inflammation, neuronal disease, and metabolic syndrome^2^. Specific granules are peroxidase-negative and contain neutrophil gelatinase-associated lipocalin (NGAL)^4^. NGAL is a known biomarker of acute kidney injury, but high levels that relate with lower estimated glomerular filtration rate have been described in chronic kidney disease^5^. Further, it has been reported that NGAL is related to several other conditions, such as arterial hypertension, obesity, and diabetes^5^.

The eosinophil granulocyte has long been known to contribute to the defense against parasitic infections. Interest in its function in inflammation linked to eosinophil-related conditions has increased markedly in the last few years with large interest in asthma^6^. Like the neutrophil, it is granulated and contains a range of proteins^7^. Eosinophil-derived neurotoxin (EDN) has been described to have a significant role within the immune response to pathogens in antiviral defense^8^ and activation of dendritic cells^9^. The latter makes it a biomarker of high interest, as the dendritic cell is important in type 2-inflammation and allergic asthma^9^. Eosinophil cationic protein (ECP) has structural similarities with EDN, and is involved in the antiviral defense, but possesses helminth toxicity and an antibacterial function. Unlike EDN, it does not appear to attract or activate dendritic cells^8^. EDN and ECP are involved in inflammation, remodeling of airways, mucus hypersecretion, and lung epithelium damage^10^. Additionally, both EDN and ECP have been described as potentially promoting atherogenesis and calcification of smooth muscle cells^11^.

The mast cell, a long-lived human cell, is involved in pathogen defense, especially at barrier locations. Through mediators, it induces an inflammatory response that may battle infections. It can also be involved in inflammation without pathogens, which can be either immunoglobulin E (IgE)-mediated or non-IgE-mediated^12^. The mast cell has granules containing the protein tryptase and others^13^. Tryptase, a protease, can be released upon strong activation signals. Serum tryptase is an established biomarker for mastocytosis and anaphylaxis, and it has also been described as adding value in analyses of kidney disease, atherosclerosis, and metabolic syndrome^14^.

In summary, the existing literature focuses on these cell activation markers in specific pathologies: NGAL in renal disorders, EDN and ECP mostly in the setting of asthma, and tryptase primarily in relation to anaphylaxis and mastocytosis. They have rarely been analyzed in large population-based studies. Therefore, we analyzed five inflammation biomarkers (NGAL, MPO, EDN, ECP, tryptase) in relation to age, sex, body mass index (BMI), chronic airflow limitation (CAL), smoking, atopy, and chronic disease in a population-based study.

## Method

### Study design and population

**Fig. 1:**
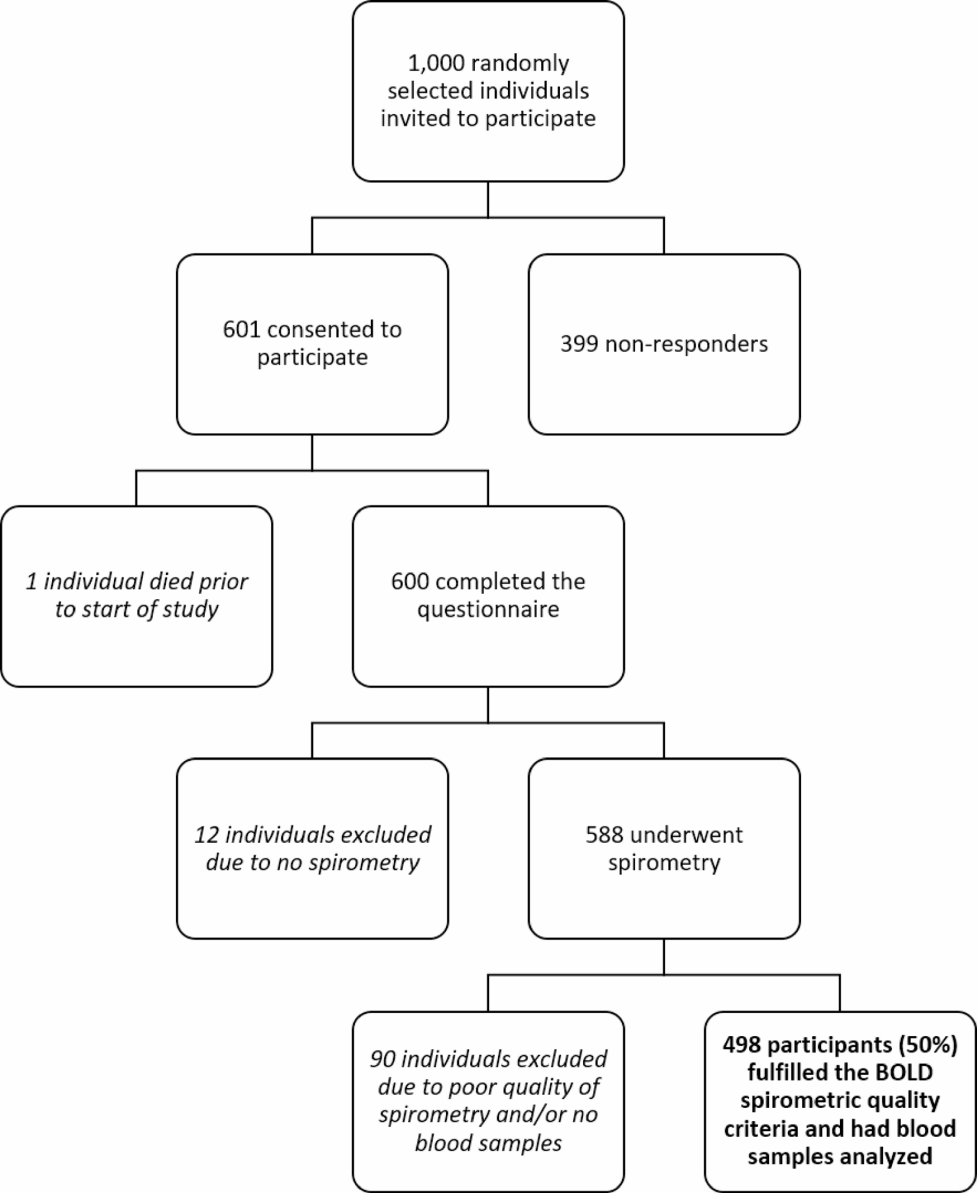
Flowchart of the inclusion process in this study.

Randomly selected individuals aged ≥ 40 years were invited to participate in the Burden of Obstructive Lung Disease (BOLD) study^15^. This is a large, international, multicenter study aiming to assess the worldwide prevalence of CAL – a defining characteristic of chronic obstructive pulmonary disease (COPD) – and identify its main risk factors^16^. The present analyses are based on the Uppsala participants of the BOLD study, with data acquired between July 2006 and December 2007. A total of 600 of the 1,000 invited individuals completed a questionnaire with background characteristics and self-reported chronic diseases. In all, 102 individuals were excluded as their spirometry data and/or blood samples were lacking. Therefore, the final number of participants was 498 (50%). A flowchart of the inclusion process is presented in Fig. 1. We have previously reported results from this population regarding EDN in relation to asthma and fixed airflow obstruction^17^.

## Questionnaire and data collection

The questionnaire included questions on, e.g., age, sex, smoking status (never-smoker/ex-smoker/current smoker), respiratory symptoms, lung diseases, other medical conditions, and medication. This is based on the European Community Respiratory Health Survey Questionnaire (ECRHS), validated and widely used^18^. Trained interviewers conducted structured interviews with the participants based on the questionnaire. Regarding self-reported doctor-diagnosed medical conditions, the participants were asked “Has a doctor ever told you that you have …?” The variable “chronic bronchitis” was based on responses in the questionnaire regarding an item on phlegm and cough for at least 3 months/year during at least 2 years.

Using an NDD EasyOne^®^ (ndd Medizintechnich, Zurich, Switzerland) spirometer, forced expiratory volume in 1 s (FEV_1_) and forced vital capacity (FVC) values were measured with the subject seated and wearing a nose clip. Pre- and post-bronchodilator tests were performed. CAL was defined as a postbronchodilator FEV_1_/FVC < 0.70. In accordance with the BOLD protocol, participants who had recently experienced a respiratory infection were asked to delay participation for 4 weeks^15^.

Height and weight were measured. BMI was calculated as weight in kilograms divided by height in meters squared.

## Measurement methods

All serum analyses were done using ImmunoCAP™ assays based on fluorescence enzyme immunoassay technology. The ImmunoCAP ECP and tryptase assays are commercially available (Thermo Fisher Scientific/Phadia AB, Uppsala, Sweden). Serum EDN, MPO, and NGAL were measured using in-house research assays described elsewhere^19,20^. ImmunoCAP Phadiatop™ (Thermo Fisher Scientific/Phadia AB) was used to measure IgE sensitization. The test encompasses a mix of common aeroallergens^21^and values are reported as Phadia arbitrary units per liter (PAU/L). Atopy was defined as having IgE antibody levels ≥ 0.35 PAU/L.

## Statistical analyses

Descriptive statistics in the forms of n (%) for categorical variables, means +/- standard deviations for normally distributed continuous variables, and geometric means (95% confidence intervals) for right-skewed distributed continuous variables (all biomarkers) were employed. For one participant, we did not obtain measurable ECP levels. Therefore, the value was set at the lowest measurable value divided by the square root of 2.

The associations between the categorical variables of interest (sex, medical conditions, etc.) and the levels of each of the five studied biomarkers were studied using unpaired t-tests performed with a log-transformed biomarker as the outcome.

Lastly, multiple linear regression models were created with log-transformed biomarkers as outcomes and predictors identified in the univariate analyses as having p-values < 0.10. The regression coefficients were back-transformed through anti-log transformation, to yield a percentual increase of biomarkers in subjects with versus without the condition of interest.

Associations with a p-value < 0.05 were considered statistically significant. All statistical analyses were performed using STATA IC 16.1 (StataCorp LLC, College Station, TX, USA).

## Ethics

The Regional Ethical Review Board in Uppsala, Sweden, approved the study for Uppsala participants 31 May 2006 (Dnr 2006/146).

The participants were provided with written information, and written informed consent was obtained from them. All methods were carried out in accordance with relevant guidelines and regulations.

## Results

The number of subjects in this study was 498, and their characteristics are presented in Table 1. The mean age was 58 years, approximately half of the participants were males, 14% were current smokers, and 15% had CAL. Hypertension was the most common medical condition (29% of participants), and 26% had atopy. For more details and levels, see Table 1.

**Table 1:** Population description.

Participants’ characteristics	(*n* = 498)
Age (years)	58 ± 11
Male sex	252 (51%)
BMI (kg/m^2^)	27.1 ± 4.4
BMI ≥ 30 kg/m^2^	107 (21%)
FEV_1_/FVC	0.77 ± 0.07
CAL	76 (15%)
Current smoker	70 (14%)
Chronic bronchitis *	17 (3%)
Medical conditions (self-reported, doctor-diagnosed) and medication	
Hypertension	142 (29%)
Heart disease	51 (10%)
Stroke	5 (1%)
Diabetes mellitus	19 (4%)
COPD, chronic bronchitis, or emphysema	24 (5%)
Current asthma	42 (8%)
Any use of Inhaled corticosteroids during the previous year	43 (9%)
Laboratory results	
Positive Phadiatop (≥ 0.35 PAU/L), S-	131 (26%)
MPO, S-, µg/L	110.4 (105.1–116.0)
NGAL, S-, µg/L	129.8 (125.9–133.9)
EDN, S-, µg/L	19.5 (18.4–20.6)
ECP, S-, µg/L	8.2 (7.7–8.7)
Tryptase, S-, µg/L ^#^	6.1 (5.9–6.3)

Values: n (%), mean values ± SD, geometric means (95% confidence intervals).

BMI = body mass index, FEV_1_ = forced expiratory volume in 1 s, FVC = forced vital capacity, CAL = chronic airflow limitation, defined as forced expiratory volume in 1 s/forced vital capacity ratio < 0.70, COPD = chronic obstructive pulmonary disease, PAU = Phadia arbitrary units, S = serum, MPO = myeloperoxidase, NGAL = neutrophil gelatinase-associated lipocalin, EDN = eosinophil-derived neurotoxin, ECP = eosinophil cationic protein.

* Based on questionnaire responses (phlegm and cough for at least 3 months/year during at least 2 years).

^#^ 2 missing values.

**Fig. 2:**
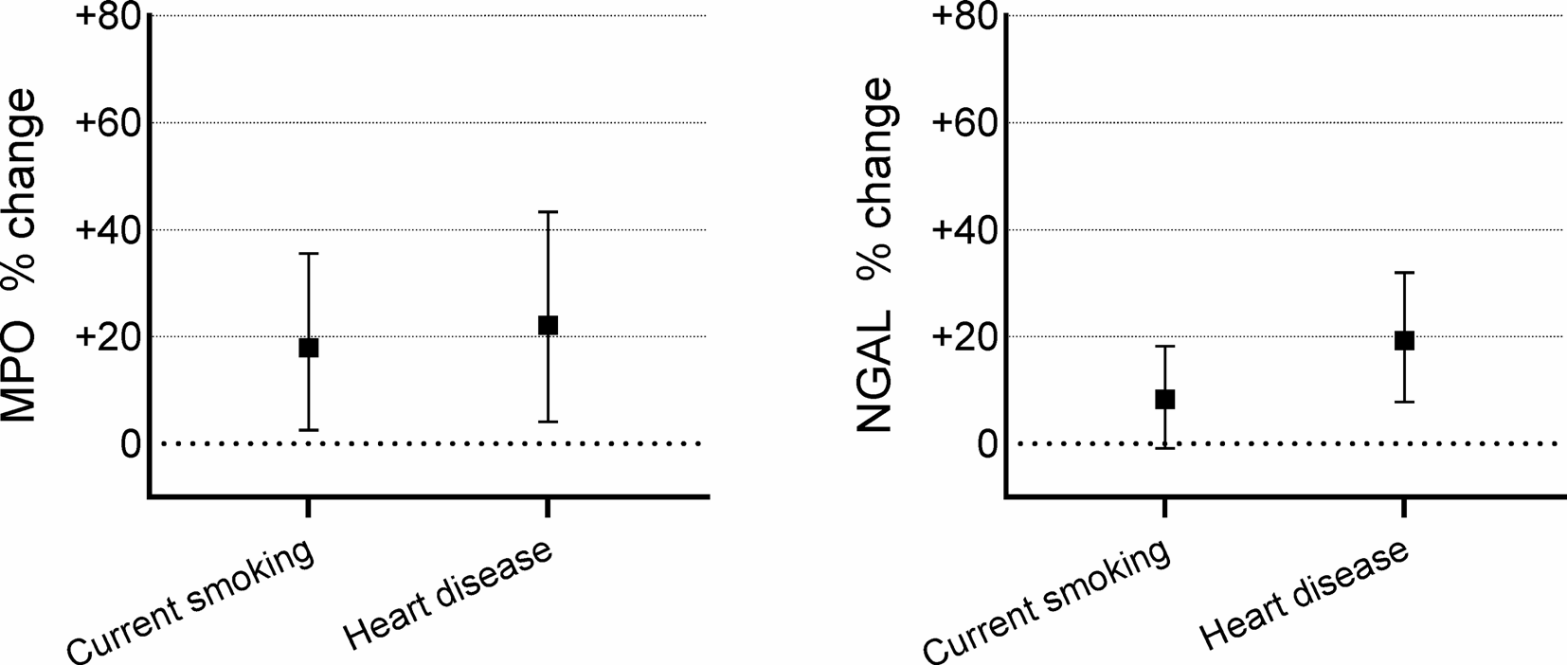
Percentual change for myeloperoxidase (MPO) and neutrophil gelatinase-associated lipocalin (NGAL) in relation to factors that had a p-value < 0.1 in univariate analyses. Beta coefficients from multivariate linear regression models were used to calculate percentual change in biomarkers for each factor.

For MPO, we found higher serum levels among those with current smoking (126.8 versus 107.9 µg/L; *p* = 0.02) and heart disease (131.6 versus 108.2 µg/L; *p* = 0.02) in univariate analyses (Table 2). These findings were confirmed in multivariate linear regression analyses, where current smoking was associated with 17.9% (95% confidence interval 2.5%, 35.6%) higher MPO levels and heart disease with 22.1% (4.1%, 43.3%) higher MPO levels (Fig. 2).

**Table 2:** Serum MPO and NGAL levels (µg/L) as a function of age, sex, BMI, chronic airflow limitation, smoking, medical conditions, and atopy.

	MPO (*n* = 498)			NGAL (*n* = 498)		
	Yes	No	p*-*value	Yes	No	p*-*value
Age ≥ 58 years (*n* = 258)	107.1 (99.9–114.9)	114.1 (106.5–122.1)	0.21	131.8 (125.6–138.2)	127.8 (122.9–132.9)	0.33
Male sex (*n* = 252)	111.0 (103.6–118.9)	109.8 (102.4–117.8)	0.83	132.3 (126.3–138.5)	127.4 (122.2–132.8)	0.24
BMI ≥ 30 kg/m^2^ (*n* = 107)	114.7 (102.8–127.9)	109.3 (103.5–115.5)	0.44	134.5 (127.4–142.1)	128.7 (124.0–133.4)	0.25
CAL (*n* = 76)	113.9 (100.7–128.8)	109.8 (104.1–115.8)	0.60	135.2 (124.9–146.4)	128.9 (124.6–133.3)	0.28
Current smoking (*n* = 70)	126.8 (111.4–144.2)	107.9 (102.4–113.8)	**0.02**	138.6 (129.1–148.8)	128.5 (124.1–132.9)	*0.09*
Chronic bronchitis* (*n* = 17)	117.0 (94.6–144.6)	110.2 (104.8–115.9)	0.66	147.6 (98.8–220.7)	129.3 (125.5–133.1)	0.13
Hypertension (*n* = 142)	105.6 (95.6–116.6)	112.4 (106.3–118.9)	0.26	132.2 (123.3–141.9)	128.9 (124.7–133.3)	0.46
Heart disease (*n* = 51)	131.6 (111.2–155.8)	108.2 (102.8–113.9)	**0.02**	151.8 (131.3–175.5)	127.5 (123.8–131.5)	**< 0.001**
Stroke (*n* = 5)	88.1 (64.9–119.6)	110.7 (105.3–116.3)	0.36	130.5 (97.8–174.3)	129.8 (125.8–134.0)	0.97
Diabetes mellitus (*n* = 19)	99.9 (76.3–130.8)	110.8 (105.4–116.5)	0.42	136.3 (120.3–154.4)	129.6 (125.5–133.8)	0.54
COPD, chronic bronchitis, or emphysema (*n* = 24)	120.7 (96.8–150.5)	109.9 (104.5–115.6)	0.42	135.5 (117.6–156.2)	129.6 (125.5–133.8)	0.54
Current asthma (*n* = 42)	112.8 (95.5–133.1)	110.2 (104.7–116.0)	0.80	131.0 (118.9–144.4)	129.7 (125.6–134.1)	0.86
Atopy (*n* = 131)	106.5 (96.7–117.3)	111.8 (105.6–118.4)	0.39	125.1 (118.7–131.9)	131.6 (126.7–136.6)	0.16

Note: Serum MPO and NGAL levels (µg/L) are presented as geometric means and 95% confidence intervals. The number of participants with each characteristic is indicated.

p-values < 0.05 are shown in bold, p-values 0.05–0.10 are shown in italics.

Abbreviations: MPO = myeloperoxidase, NGAL = neutrophil gelatinase-associated lipocalin, BMI = body mass index, CAL = chronic airflow limitation, defined as forced expiratory volume in 1 s/forced vital capacity ratio < 0.70, COPD = chronic obstructive pulmonary disease.

*Based on questionnaire responses (phlegm and cough for at least 3 months/year during at least 2 years).

Higher levels of NGAL were found in subjects with heart disease (151.8 versus 127.5 µg/L; *p* < 0.001) in univariate analyses (Table 2). Subjects with heart disease had 19.3% (7.8%, 31.9%) higher NGAL levels than subjects without heart disease, after adjustment for current smoking (Fig. 2).

**Fig. 3:**
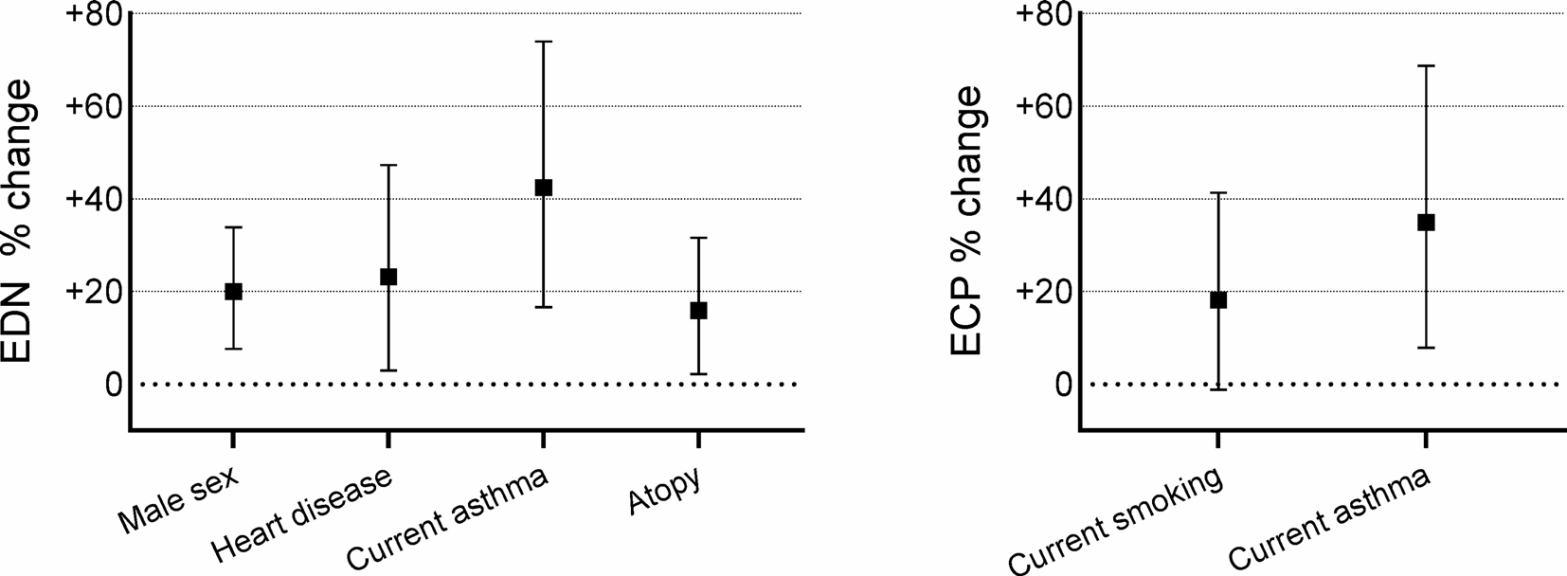
Percentual change for eosinophil-derived neurotoxin (EDN) and eosinophil cationic protein (ECP) in relation to factors that had a p-value < 0.1 in univariate analyses. Beta coefficients from multivariate linear regression models were used to calculate percentual change in biomarkers for each factor.

Higher EDN levels were related to male sex, heart disease, current asthma, and atopy, with the highest levels found in relation to current asthma (27.8 versus 18.9 µg/L; *p* < 0.001) (Table 3). In multivariate linear regression analyses, subjects with current asthma were found to have 42.5% (16.7%, 74.0%) higher EDN levels than subjects without current asthma and subjects with heart disease were found to have 23.2% (3.0%, 47.3%) higher EDN levels than subjects without heart disease (Fig. 3). Similarly, male sex and atopy were consistently related with higher EDN levels in multivariable linear regression analyses (Fig. 3). When BMI was analyzed as a continuous variable, a trend towards association with EDN was found in univariate analyses (*p* = 0.06), but the relation was weaker (*p* = 0.14) in the multivariate linear regression model. Furthermore, the association between EDN and current asthma was consistent (*p* = 0.001) after adjusting for continuous BMI (data not shown). Higher ECP levels were found in subjects with current asthma than those without (10.7 versus 8.1 µg/L; *p* = 0.01) (Table 3). Multivariate linear regression analyses showed 34.9% (7.8%, 68.7%) higher ECP levels in subjects with current asthma than those without, even after adjustment for current smoking (Fig. 3). Finally, no significant interactions with use of inhaled corticosteroids during the previous year were found on the association between current asthma and EDN (*p* = 0.96) nor on the association between current asthma and ECP (*p* = 0.27).

**Table 3:** Serum EDN and ECP levels (µg/L) as a function of age, sex, BMI, chronic airflow limitation, smoking, medical conditions, and atopy.

	EDN (*n* = 498)			ECP (*n* = 498)		
	Yes	No	p-value	Yes	No	p-value
Age ≥ 58 years (*n* = 258)	19.8 (18.3–21.5)	19.2 (17.7–20.7)	0.56	8.2 (7.5–9.0)	8.2 (7.6–9.0)	0.85
Male sex (*n* = 252)	21.4 (19.8–23.2)	17.7 (16.4–19.2)	**< 0.001**	8.4 (7.7–9.2)	8.1 (7.4–8.8)	0.66
BMI ≥ 30 kg/m^2^ (*n* = 107)	20.5 (18.0–23.4)	19.3 (18.1–20.5)	0.37	8.6 (7.4–10.0)	8.2 (7.6–8.7)	0.44
CAL (*n* = 76)	21.7 (18.9–25.0)	19.1 (18.0–20.3)	0.11	8.5 (7.4–9.7)	8.2 (7.7–8.8)	0.67
Current smoking (*n* = 70)	20.8 (18.1–24.0)	19.3 (18.2–20.5)	0.35	9.4 (8.2–10.9)	8.1 (7.5–8.6)	*0.08*
Chronic bronchitis* (*n* = 17)	21.9 (14.2–33.8)	19.4 (18.4–20.6)	0.44	7.1 (4.8–10.7)	8.3 (7.8–8.8)	0.42
Hypertension (*n* = 142)	19.5 (17.5–21.7)	19.5 (18.3–20.8)	0.98	7.9 (7.0–8.9)	8.4 (7.8–9.0)	0.46
Heart disease (*n* = 51)	23.5 (19.6–28.2)	19.1 (18.0–20.3)	**0.03**	9.5 (7.8–11.6)	8.1 (7.6–8.7)	0.11
Stroke (*n* = 5)	19.8 (9.8–40.1)	19.5 (18.4–20.6)	0.96	5.6 (3.0–10.1)	8.3 (7.8–8.8)	0.22
Diabetes mellitus (*n* = 19)	23.6 (17.1–32.6)	19.4 (18.3–20.5)	0.18	10.3 (7.6–14.0)	8.2 (7.7–8.7)	0.15
COPD, chronic bronchitis, or emphysema (*n* = 24)	18.8 (14.5–24.3)	19.5 (18.5–20.7)	0.77	9.5 (7.2–12.6)	8.2 (7.7–8.7)	0.30
Current asthma (*n* = 42)	27.8 (23.2–33.3)	18.9 (17.8–20.0)	**< 0.001**	10.7 (8.7–13.2)	8.1 (7.5–8.6)	**0.01**
Atopy (*n* = 131)	22.7 (20.4–25.4)	18.5 (17.3–19.7)	**0.001**	9.0 (8.0–10.1)	8.0 (7.4–8.6)	0.21

Note: Serum EDN and ECP levels (µg/L) are presented as geometric means and 95% confidence intervals. The number of participants with each characteristic is indicated.

p-values < 0.05 are shown in bold, p-values 0.05–0.10 are shown in italics.

Abbreviations: EDN = eosinophil-derived neurotoxin, ECP = eosinophil cationic protein, BMI = body mass index, FEV_1_ = forced expiratory volume in 1 s, FVC = forced vital capacity, CAL = chronic airflow limitation, defined as forced expiratory volume in 1 s/forced vital capacity ratio < 0.70, COPD = chronic obstructive pulmonary disease.

*Based on questionnaire responses (phlegm and cough for at least 3 months/year during at least 2 years).

Higher levels of tryptase were found in obese subjects (BMI ≥ 30 kg/m^2^), subjects with CAL, and subjects with hypertension, with the highest concentrations found in relation to obesity (6.9 versus 5.9 µg/L; *p* = 0.002) and CAL (6.9 versus 6.0 µg/L; *p* = 0.007) (Table 4).

**Table 4:** Serum tryptase levels (µg/L) as a function of age, sex, BMI, chronic airflow limitation, smoking, medical conditions, and atopy.

	Tryptase (*n* = 496)		
	Yes	No	p-value
Age ≥ 58 years (*n* = 257)	6.3 (6.0–6.7)	5.9 (5.6–6.2)	*0.09*
Male sex (*n* = 251)	6.2 (5.9–6.5)	6.0 (5.7–6.4)	0.45
BMI ≥ 30 kg/m^2^ (*n* = 106)	6.9 (6.4–7.4)	5.9 (5.7–6.2)	**0.002**
CAL (*n* = 76)	6.9 (6.2–7.6)	6.0 (5.7–6.2)	**0.007**
Current smoking (*n* = 70)	6.5 (5.8–7.2)	6.1 (5.8–6.3)	0.21
Chronic bronchitis* (*n* = 16)	6.5 (4.7–9.1)	6.1 (5.9–6.3)	0.53
Hypertension (*n* = 140)	6.5 (6.0–7.1)	6.0 (5.7–6.2)	**0.02**
Heart disease (*n* = 50)	6.4 (5.7–7.2)	6.1 (5.8–6.3)	0.43
Stroke (*n* = 5)	8.2 (5.7–11.8)	6.1 (5.9–6.3)	0.12
Diabetes mellitus (*n* = 18)	6.4 (5.7–7.1)	6.1 (5.9–6.3)	0.66
COPD, chronic bronchitis, or emphysema (*n* = 24)	5.8 (4.8–7.1)	6.1 (5.9–6.4)	0.58
Current asthma (*n* = 42)	6.0 (5.4–6.8)	6.1 (5.9–6.4)	0.80
Atopy (*n* = 129)	5.9 (5.6–6.3)	6.2 (5.9–6.5)	0.30

Note: Serum tryptase levels (µg/L) are presented as geometric means and 95% confidence intervals. The number of participants with each characteristic is indicated.

p-values < 0.05 are shown in bold, p-values 0.05–0.10 are shown in italics.

Abbreviations: BMI = body mass index, FEV_1_ = forced expiratory volume in 1 s, FVC = forced vital capacity, CAL = chronic airflow limitation, defined as forced expiratory volume in 1 s/forced vital capacity ratio < 0.70, COPD = chronic obstructive pulmonary disease.

*Based on questionnaire responses (phlegm and cough for at least 3 months/year for at least 2 years).

BOLD = Burden of Obstructive Lung Disease.

**Fig. 4:**
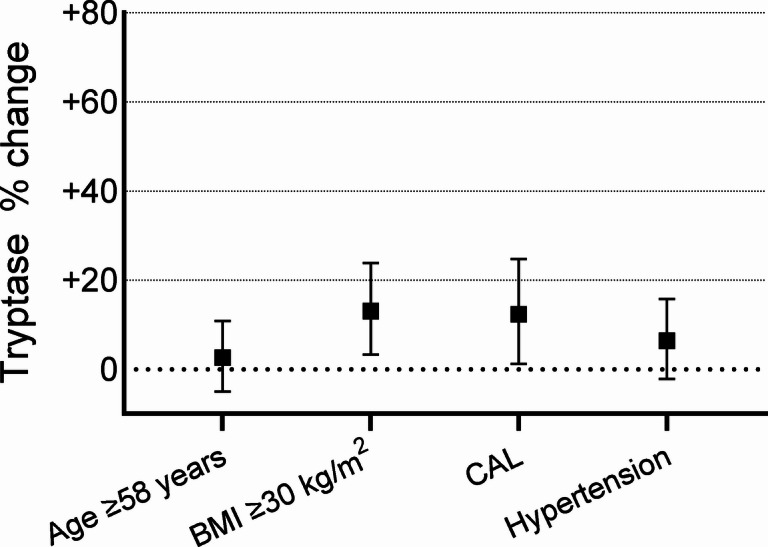
Percentual change for tryptase in relation to age, body mass index ≥ 30 kg/m^2^, chronic airflow limitation (CAL) and hypertension (all p-value < 0.1 in univariate analyses). Beta coefficients from multivariate linear regression models used to calculate percentual change in biomarker for each factor.

After adjusting for age (≥ 58 years), obesity, CAL, and hypertension in a multivariate linear regression analysis, obesity related to 13.1% (3.3%, 23.9%) higher tryptase and CAL to 12.3% (1.2%, 24.7%) higher tryptase, whereas the relation with hypertension became non-significant (Fig. 4). When BMI was analyzed as a continuous variable instead of being dichotomized at 30 kg/m², it remained statistically significantly associated with tryptase, in both univariate (*p* = 0.006) and multivariate linear regression analyses (*p* = 0.02). Age remained statistically significant associated with tryptase in both univariate and multivariate linear regression analyses (both *p* ≤ 0.001) when assessed as a continuous variable rather than dichotomized at 58 years. When BMI and age were both included as continuous variables CAL was no longer significantly associated with tryptase level in the multivariate linear regression analyses (*p* = 0.16) (data not shown).

## Discussion

We made several key findings related to each biomarker. MPO and NGAL – activation markers for neutrophil granulocytes – were associated with heart disease. MPO was also associated with current smoking, and a similar trend was seen for NGAL. Higher levels of EDN were independently related to male sex, heart disease, current asthma, and atopy. We found a positive association between ECP and current asthma. Higher levels of tryptase related to obesity.

The MPO and NGAL findings are similar, which seems reasonable as both originate mainly from the neutrophil granulocyte. NGAL is also found in other cells (macrophages, dendritic cells) and various human tissues (such as kidney, heart, lung, liver, and the vascular system)^5^ and MPO can also be found in monocytes^22^. The relationships of MPO and NGAL to heart disease have been described previously^23–27^ mainly for heart failure and coronary heart disease^23–25,27^. Moreover, higher NGAL levels have been related to increased mortality over a 2-year period in elderly patients with chronic heart failure^28^ and both higher NGAL levels and higher MPO levels have been related to higher rates of major adverse cardiovascular events in patients with acute coronary syndrome^29^. Higher levels of MPO in peripheral blood have also been described as being associated with atrial fibrillation^25,26^. In our study, we were unable to distinguish between various heart conditions. Though there appear to be few publications, associations between smoking and MPO and NGAL levels are mentioned in the literature^30,31^. As smoking increases blood neutrophil counts^32^ and cigarette smoke induces systemic effects and inflammation^33,34^ this seems to be a reasonable finding.

An association between higher concentrations of EDN in peripheral blood and male sex has previously been shown in both children^35^ and adults^10^ and corresponds with our observation. However, in one study of 120 individuals, no significant differences were found between sexes^36^. The finding regarding EDN and heart disease is interesting and in line with the results of a recent Swedish population-based study in a narrow age range, 50–64 years, where a relation was found with angina pectoris but not heart failure^37^. Due to the cross-sectional nature of both studies, it is difficult to draw any conclusions regarding causality. EDN could promote atherogenesis and calcification of smooth muscle cells^11^. An association with heart disease has previously been reported for serum ECP^38^. However, we could not confirm these results in our study. A link between higher levels of EDN and heart disease seems reasonable as both EDN and ECP originate from the same cell, and the eosinophil has been described to be involved in heart disease^39–41^. EDN and ECP are well-described in asthma (8, 31), and our study could confirm this correlation. We observed a trend toward higher ECP levels with current smoking, which aligns with previous observations by Jensen et al.^42^ and findings on eosinophil counts from population-based studies^43^.

Obese individuals had higher tryptase levels, a finding in line with a previous report^44^ but this has not been thoroughly investigated. We found initially a relation between CAL and higher tryptase levels, but this was not consistent in the adjusted model for continuous age and BMI. A previous, larger study could not find a significant association between CAL and tryptase in a middle-aged population^14^.

This study’s strengths include that it used a population-based cohort with a broad age range and that several variables and medical conditions were examined. It might be regarded as a limitation for external validity that the data were collected more than 15 years ago, as changes in smoking habits and BMI have been seen in the general population during that time. Moreover, it could be argued that the stability of these biomarkers might be affected by the long storage time of the samples although Brussal et al. reported that tryptase has a very a good long-term stability over a 8-years’ period^45^. However, as we looked mainly at correlations – not the prevalence of elevated levels of biomarkers or potential cut-off values – we believe the current findings are valid. Another limitation was that we could not separate types of heart disease, such as heart failure, ischemic heart disease, or arrhythmias. Additional limitations can include the unknown timing of the illnesses and the low prevalence of some conditions, including stroke, which limit the statistical power of the respective analyses. Therefore, the absence of correlations for rare conditions cannot exclude that such associations would exist in more extensive materials. Moreover, as the study was cross-sectional, we could not investigate causality, only associations between biomarkers and disease. A further limitation of this study is the absence of detailed information on dyslipidemia, including both diagnoses and the use of lipid-lowering therapies. This is relevant given that dyslipidemia is a well-established risk factor for atherosclerosis, and the biomarkers investigated in this study are associated with atherosclerotic processes. Similarly, lack of information on renal function is a limitation as NGAL is related to kidney function. Finally, we had only information on respiratory medication, but no information on medication for cardiometabolic disease.

## Conclusions

In this exploratory study based on a population of 498 randomly selected individuals aged ≥ 40 years, we could identify conditions associated with activity markers for neutrophil, eosinophil, and mast cell activation in serum. Some outcomes are more acknowledged, such as EDN and ECP in asthma and the correlation between MPO and NGAL in heart disease. Still, the finding of higher EDN levels in individuals with heart disease is novel. This needs to be further confirmed in other populations and its potential clinical use remains to be established.

## Data Availability

Due to Swedish personal data protection legislation, study data cannot be freely shared. After ethical approval, anonymized individual-level data will be made available by the corresponding author.
